# Supplementary figures and images for: A groupwise registration and tractography framework for cardiac myofiber architecture description by diffusion MRI: An application to the ventricular junctions
Source: PLoS One. 2022 Jul 18;17(7):e0271279. doi: 10.1371/journal.pone.0271279 (PMC9292118; doi:10.1371/journal.pone.0271279)

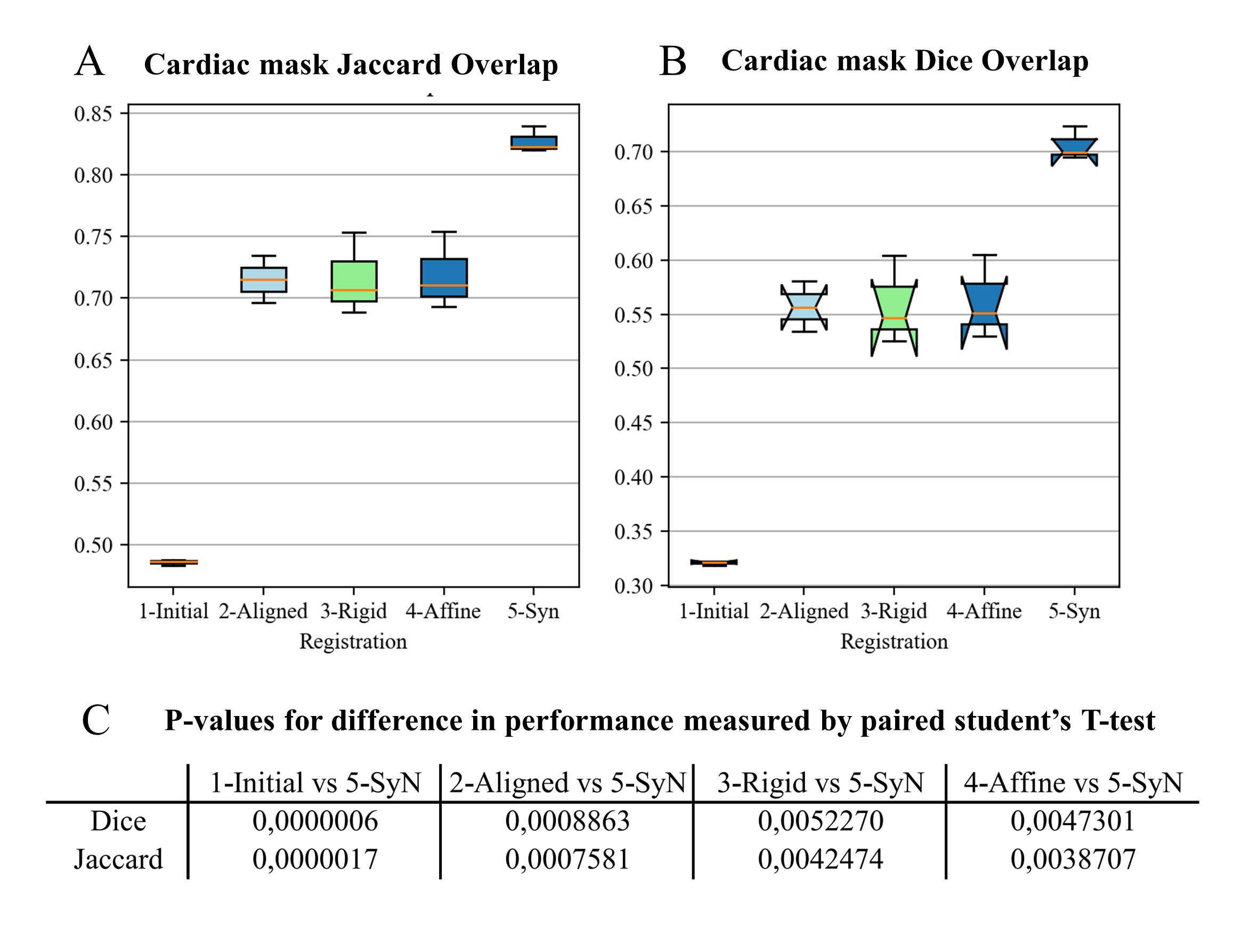

Supplement: S1 Fig — (PNG) [file pone.0271279.s001.png]

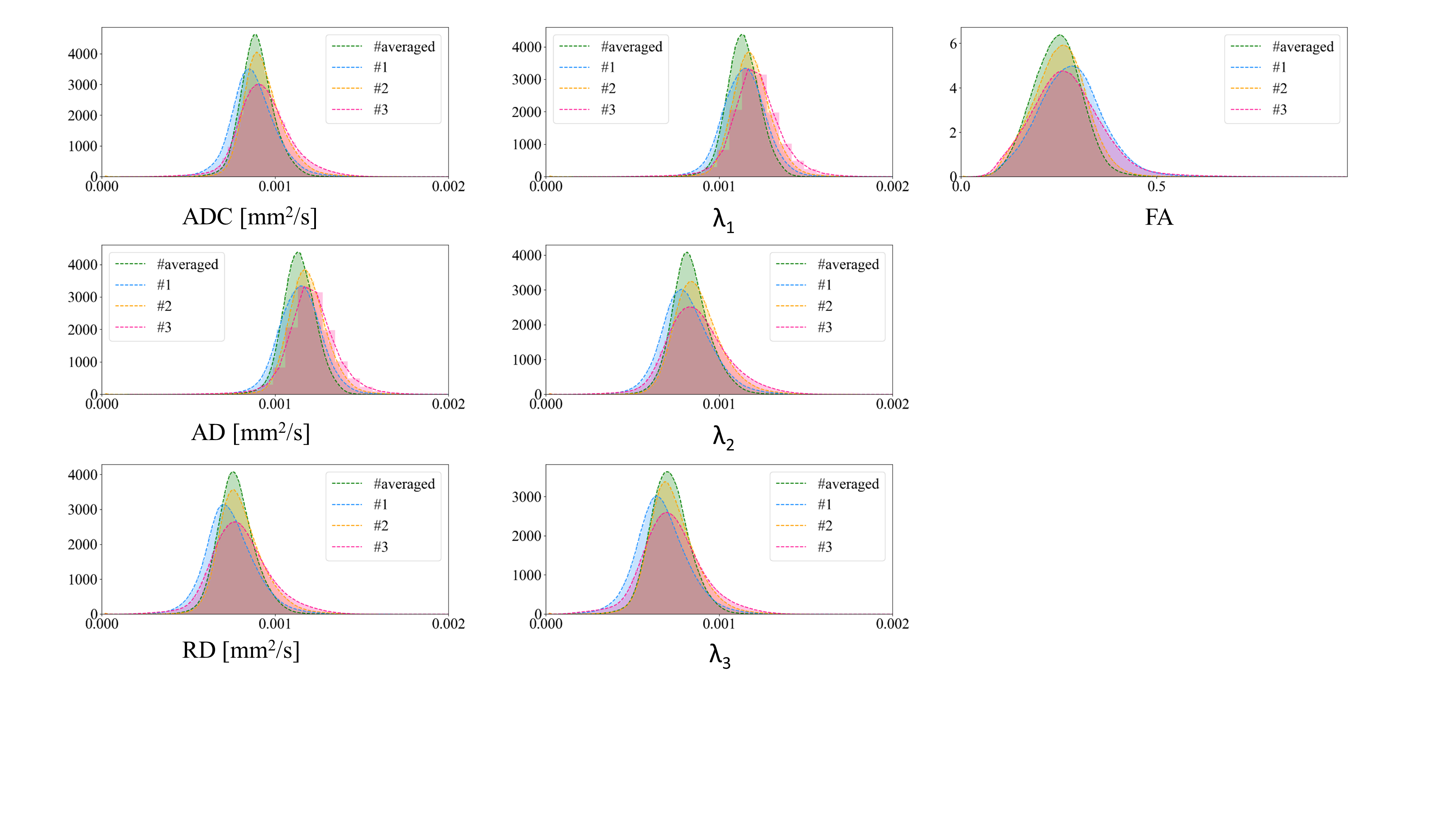

Supplement: S2 Fig — (PNG) [file pone.0271279.s002.png]

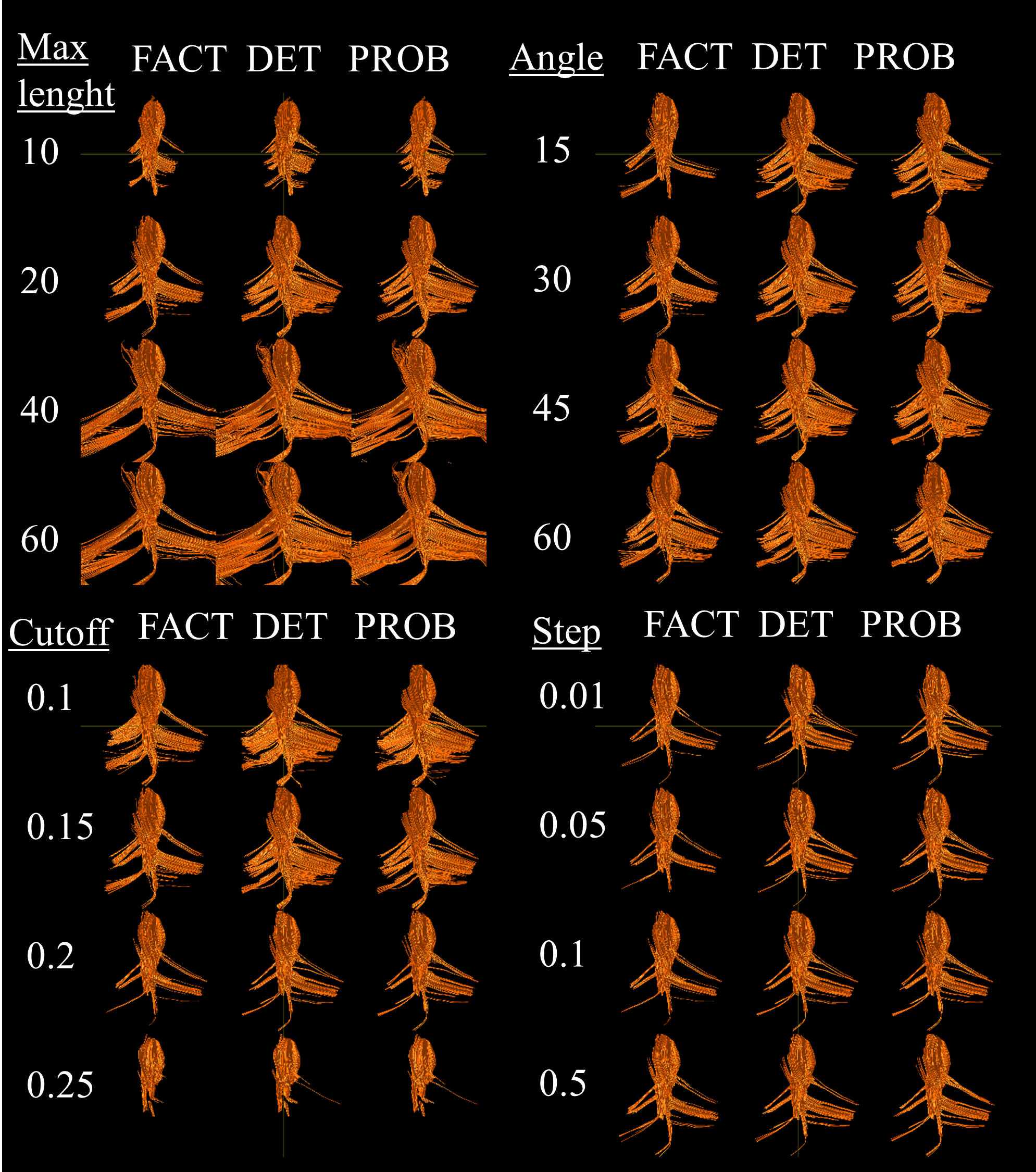

Supplement: S4 Fig — (PNG) [file pone.0271279.s004.png]

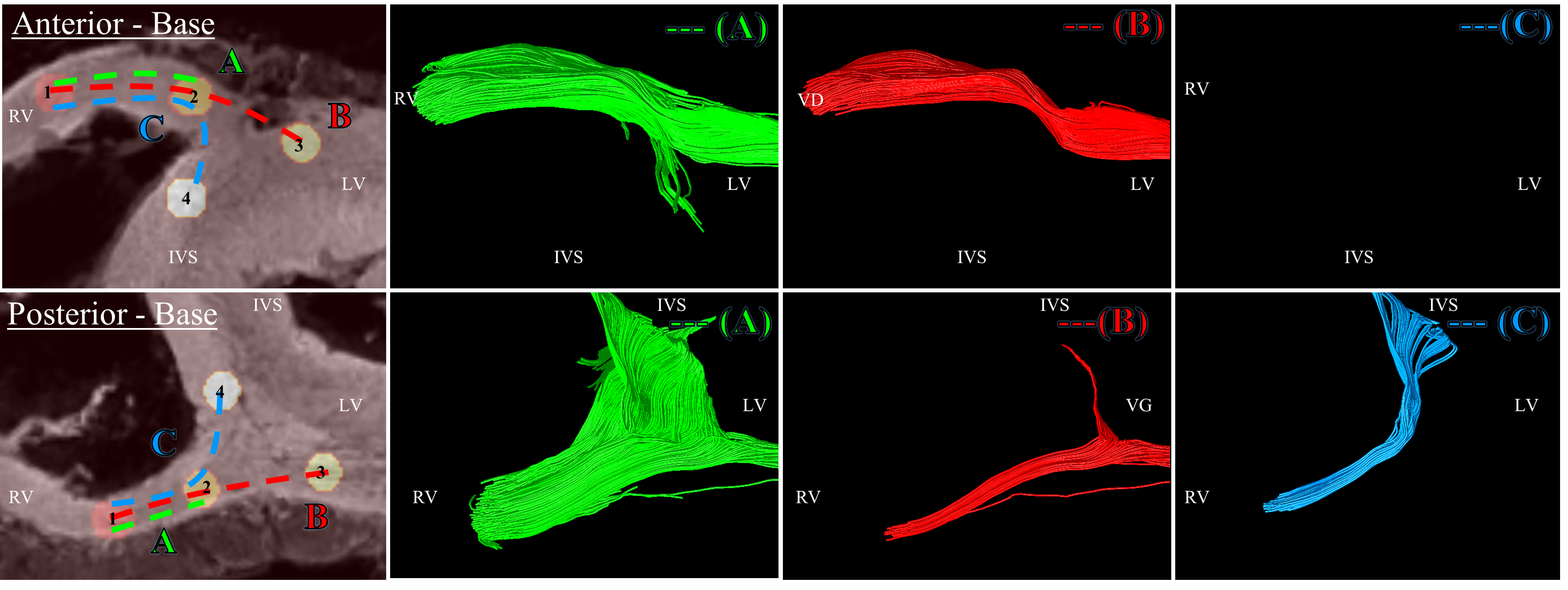

Supplement: S5 Fig — (PNG) [file pone.0271279.s005.png]

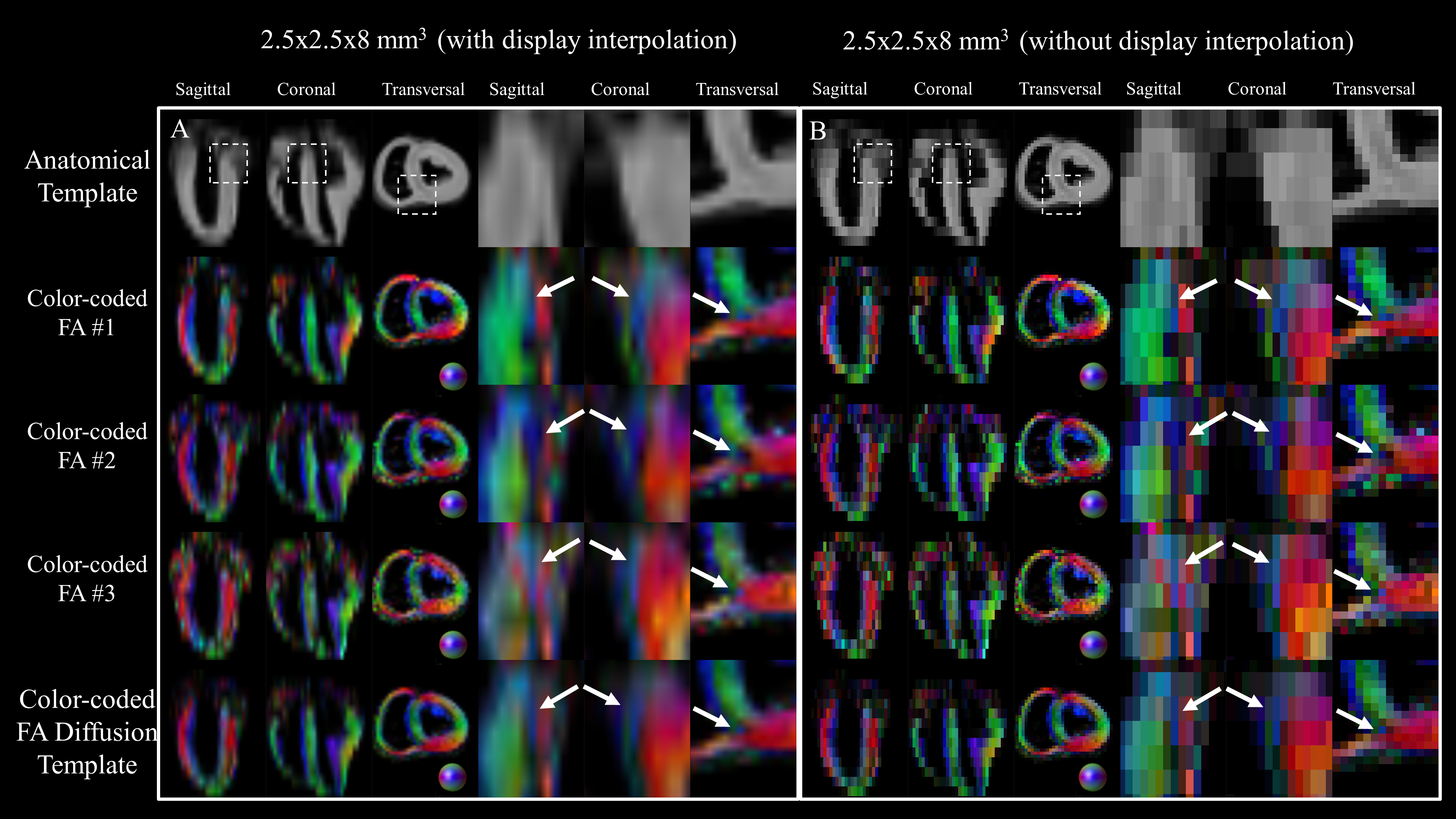

Supplement: S6 Fig — (PNG) [file pone.0271279.s006.png]

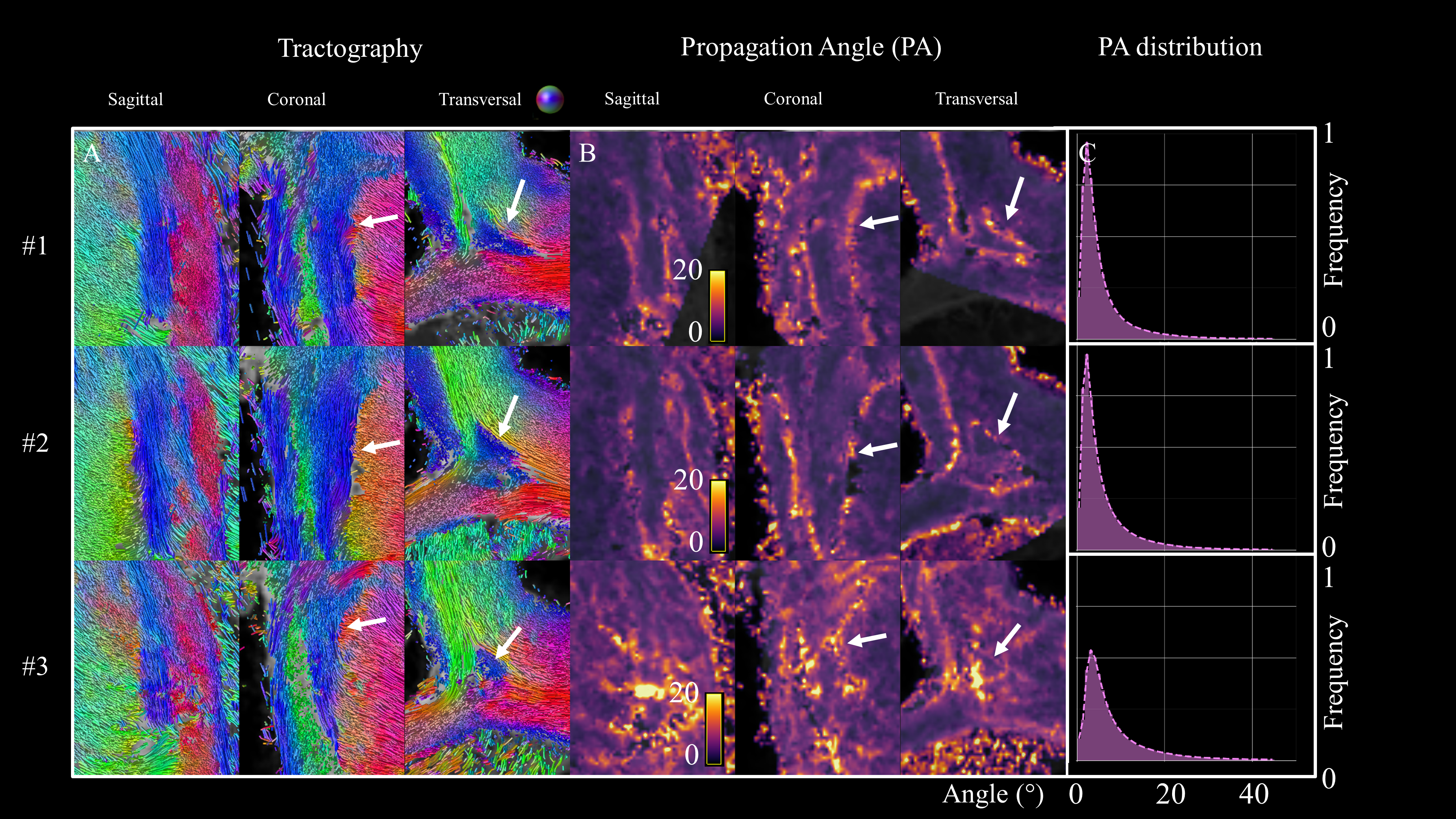

Supplement: S7 Fig — (PNG) [file pone.0271279.s007.png]
